# Cur@SF NPs alleviate Friedreich’s ataxia in a mouse model through synergistic iron chelation and antioxidation

**DOI:** 10.1186/s12951-022-01333-9

**Published:** 2022-03-09

**Authors:** Li Xu, Zichen Sun, Zhiyao Xing, Yutong Liu, Hongting Zhao, Zhongmin Tang, Yu Luo, Shuangying Hao, Kuanyu Li

**Affiliations:** 1 Jiangsu Key Laboratory of Molecular Medicine, Medical School of Nanjing University, Nanjing, 210093 China; 2 State Key Laboratory of Pharmaceutical Biotechnology, Division of Iron Metabolism and Mitochondrial Function, Medical School of Nanjing University, Nanjing, 210093 China; 3 Shanghai Institute of Ceramics, Chinese Academy of Sciences, Shanghai, 200050 China; 4 Shanghai Engineering Technology Research Center for Pharmaceutical Intelligent Equipment, Shanghai Frontiers Science Research Center for Druggability of Cardiovascular Noncoding RNA, Institute for Frontier Medical Technology, College of Chemistry and Chemical Engineering, Shanghai University of Engineering Science, Shanghai, 201620 China; 5 School of Medicine, Henan Polytechnic University, Jiaozuo, 454003 Henan China

**Keywords:** Curcumin, Silk fibroin, Drug delivery, FRDA, Mitochondrial function

## Abstract

Abnormal iron metabolism, mitochondrial dysfunction and the derived oxidative damage are the main pathogeneses of Friedrich's ataxia (FRDA), a single-gene inherited recessive neurodegenerative disease characterized by progressive cerebellar and sensory ataxia. This disease is caused by frataxin (*FXN*) mutation, which reduces FXN expression and impairs iron sulfur cluster biogenesis. To date, there is no effective therapy to treat this condition. Curcumin is proposed harboring excellent ability to resist oxidative stress through Nrf2 activation and its newly found ability to chelate iron. However, its limitation is its poor water solubility and permeability. Here, we synthesized slow-release nanoparticles (NPs) by loading curcumin (Cur) into silk fibroin (SF) to form NPs with an average size of 150 nm (Cur@SF NPs), which exhibited satisfactory therapeutic effects on the improvement of FRDA manifestation in lymphoblasts (1 μM) derived from FRDA patients and in YG8R mice (150 mg/kg/5 days). Cur@SF NPs not only removed iron from the heart and diminished oxidative stress in general but also potentiate iron-sulfur cluster biogenesis, which compensates FXN deficiency to improve the morphology and function of mitochondria. Cur@SF NPs showed a significant advantage in neuron and myocardial function, thereby improving FRDA mouse behavior scores. These data encourage us to propose that Cur@SF NPs are a promising therapeutic compound in the application of FRDA disease.

**Graphical Abstract:**

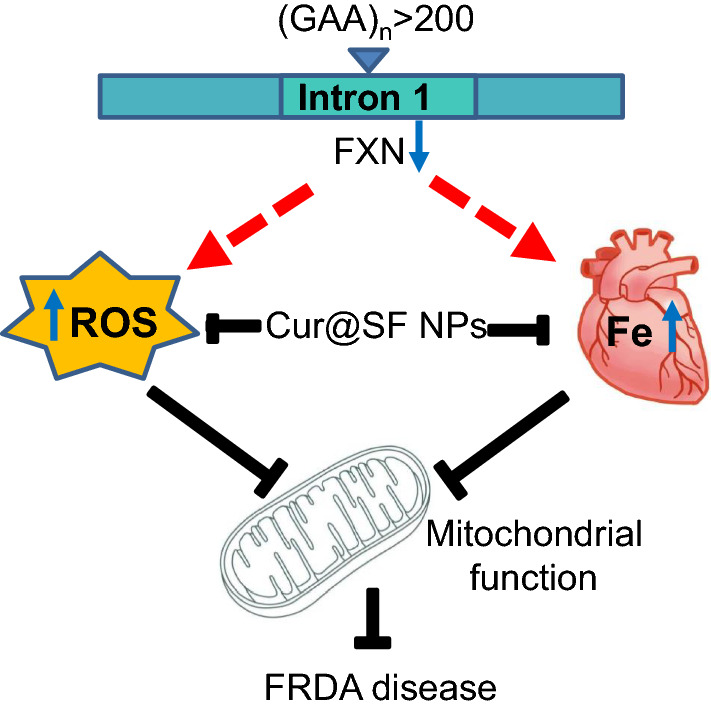

**Supplementary Information:**

The online version contains supplementary material available at 10.1186/s12951-022-01333-9.

## Background

Friedrich's ataxia (FRDA) is a single-gene inherited recessive neurodegenerative disease caused by expansion of triplet nucleotide GAA repeats in the first intron of the frataxin (*FXN*) gene, characterized by progressive cerebellar and sensory ataxia [1]. The GAA expansion mutation leads to reduction of the transcription of frataxin, a highly conserved nuclear-encoded mitochondrial protein involved in the biosynthesis of iron sulfur cluster (Fe-S) [2]. Deficiency of Fe-S compromises mitochondrial quality, including its disorganized cristae, less cristae, and less mitochondrial number, resulting in mitochondrial dysfunction (reviewed in [3]). Abnormal iron metabolism and mitochondrial dysfunction are the pathologic and cytologic basis of the disease, and the cerebellum, heart, and spine are the main affected tissues [4–6]. Of them, severe cardiomyopathy, especially hypertrophic cardiomyopathy induced by iron accumulation in mitochondria and oxidative stress, is the main cause of death in patients [7]. This cardiomyopathy in FRDA patients naturally transitions from hypertrophy to dilation, which promotes the death of cardiomyocytes and the replacement of contractile cells by fibrotic tissue, inducing severe systolic and diastolic dysfunction [8, 9].

It is believed that the accumulated iron produces toxic free radicals through the Fenton reaction, suggesting the potential therapeutic effect of iron chelators and antioxidants [10]. Iron-rich aggregates are reminiscent of mitochondrial phosphate-iron nanoparticles that were identified in mutated Yfh1 yeast [11]. Aggregated iron was also observed as mineral nonferritin aggregates in muscle creatine kinase (MCK) mice [12], a conditional *FXN* knockout mouse model reproducing the cardiac phenotype. This finding implies that iron deposits in FRDA patients are still in the form of inorganic substances. Clinically, iron chelation and antioxidative therapies are two of the main strategies for the treatment of FRDA diseases [13]. Regrettably, iron chelation, e.g., deferiprone (DFP), and direct antioxidant strategies were suspended due to side effects and no effects on the improvement of neurological scores [14, 15]. Novel strategies would be considered with the use of a cocktail of several drugs and/or the development of a compound possessing two or more active moieties that simultaneously manipulate multiple targets [16–19].

The traditional Chinese medicine curcumin has shown the effects of lowering blood lipids, antitumor, anti-inflammatory, antioxidation and treating neurodegenerative diseases as reviewed in ref [19, 20]. Importantly, the methoxy and phenolic hydroxyl structures on the benzene ring and the double carbonyl structure of curcumin are both able to chelate iron ions and a more stable curcumin-iron chelate [21]. However, the poor water solubility and poor absorption of curcumin into the gastrointestinal tract greatly reduces its utilization, and bare curcumin is more likely to be occupied by extracellular free metal ions, which will reduce the iron complexing ability after entering cells [22]. Nanomedicines offer significant advantages in free radical scavenging and body uptake [23, 24]. Therefore, some researchers have modified curcumin, such as curcumin-hydrogel [25, 26] and curcumin-graphene [27, 28], to improve the bioavailability of curcumin.

Here, we developed Cur@SF NPs by loading curcumin into silk fibroin (SF, a protein extracted from natural silk) nanospheres, taking advantage of the in vivo slow-release feature of SF [29, 30]. Cur@SF NPs exhibited a powerful effect in reducing the oxidative stress level and removing the accumulated iron in the myocardial tissue of FRDA mice. The behavioral and histological assays exhibited excellent therapeutic efficacy of Cur@SF NPs in improving neurological deficits and cardiomyopathy. Thus, we provide evidence for low-cost agent Cur@SF NPs by increasing their bioavailability, suggesting their potential in the treatment of FRDA disease.

## Materials and methods

### Preparation of regenerated silk fibroin and curcumin nanoparticles (Cur@SF NPs)

Bombyx mori silk bought from Xinyuan Co. Ltd. The regeneration of silk fibroin was performed as described previously [31]. The dialyzed silk fibroin solution was concentrated to 7%.

Curcumin was purchased from MERYER (M17074-25G). The synthetic process of curcumin-loaded SF nanoparticles (Cur@SF NPs) was followed as described previously [30]. Briefly, 10 mg curcumin was dissolved to 100 ml anhydrous ethanol. Then 10 ml of 7% silk fibroin (SF) solution was added dropwise 100 μL per minute. The mixed solution was placed in a − 20 °C freezer for 48 h. After removal of the solution by centrifugation at 20,000 *g* for 10 min, the acquired Cur@SF NPs were then collected.

### Characterization of Cur@SF NPs

The morphology of the nanoparticles was detected by scanning electron microscopy (SEM, JEM -2100, Japan) at an accelerating voltage of 15 kV after palladium sputter coating. The measurement of the nanoparticle size was carried out with a particle size potential analyzer (Malvern, Nano-ZS90). The characteristic absorption of Cur@SF NPs was analyzed (UV-3600, SHIMADZU).

### Fourier transform infrared spectroscopy (FTIR)

The SF, curcumin and Cur@SF NP solutions were deposited on 96-well IR-transparent Si plates (Bruker, Billerica, MA, USA) and dried at room temperature for at least 30 min to form dry films. FTIR measurements were performed using a High Throughput Screening eXTension (HTS-XT) unit coupled to a Tensor 27 spectrometer (Bruker, Billerica, MA, USA). The spectra were recorded in the region between 4000 and 500 cm^−1^ with a spectral resolution of 4 cm^−1^ and an aperture of 5.0 mm. For each spectrum, 40 interferograms were collected and averaged. Data acquisition was controlled using Opus v6.5 (Bruker, Billerica, MA, USA).

### Drug release rate

First, 10 mg of curcumin was dissolved in 100 ml of anhydrous ethanol to get a homogenous solution. Then 0.1, 0.2, 0.3, 0.4, and 0.5 mL of the solution was added to anhydrous ethanol to make up to 10 ml. Using anhydrous ethanol as a blank control, the absorbance values were recorded at 435 nm, and a calibration curve was drawn with absorbance (A) as Y-axis and concentration (C) as X-axis. The resulting regression equation: A = 0. 1314C + 0. 0278, r = 0. 9998 (Additional file 1: Fig. S1) (n = 3).

The drug release rate was assessed by protease XIV (Sigma UK) degradation experiments. Cur@SF NP nanoparticles (500 μg curcumin) was incubated in 5 ml of phosphate buffered saline (PBS, pH = 7.4) with protease XIV (1 U/ml at 37 °C). The enzyme solution was changed every two days. The absorbance was recorded at 435 nm, and the release rate was calculated with reference to the calibration curve.

### Labile iron pool (LIP) measurement

Labile iron was measured using iron probes calcein-AM (Aladdin, Shanghai, China) or rhodamine B-[(1,10-phenanthroline-5-yl)-aminocarbonyl]benzyl ester (RPA, Squarix GmbH, Marl, Germany) as previously reported [32]. For the cytosolic LIP, 100 µM 2,2’-bipyridyl (BIP), an iron chelator, was added to quench the calcein-iron complex. The increase in fluorescence after adding BIP was expressed as the level of LIP in the cytoplasm. For the mitochondrial LIP, the quenching of RPA by iron was revealed after the addition of the specific iron chelator pyridoxal isonicotinoyl hydrazone (PIH, final concentration 2 mM) for 30 min. The difference in the fluorescence before and after PIH chelation represents the mitochondrial LIP.

### Ferrozine iron assay

Total proteins (100 μg) were heated with 11.6 M HCl at 95 °C for 20 min and reduced with 75 mM ascorbic acid following centrifugation. Ferrozine and supersaturated ammonium acetate were added, and the absorption at 562 nm was recorded as described in [32].

### Mice and cells

Lymphoblasts derived from healthy controls (GM15849) or FRDA patients (GM15850) were purchased from the Coriell Institute for Medical Research Repository (Camden, NJ). FRDA transgenic mice YG8R (#012253) and control mice Y47 (#024097) were purchased from the Jackson laboratory (https://www.jax.org). Animals were group-housed under standard housing conditions with a 12 h light–dark cycle and temperature of 25 °C. Cur@SF NPs were injected intraperitoneally every five days at a dose of 150 mg/kg (n = 10/group). All animal experiments were reviewed and approved by the Animal Investigation Ethics Committee of Nanjing University and performed according to the Guidelines for the Care and Use of Laboratory Animals published by the National Institutes of Health, USA.

### Behavioral testing

#### Rotarod test

The motor functions of balance and coordination were assessed using an accelerating rotarod (Jiangsu SANS Technology Co., Ltd, Nanjing, China) as previously described [33].

#### Hang wire test

A metal net woven with a wire of 1 mm diameter was fixed at a position 1.5 m from the ground, and protective items were placed directly underneath to prevent the mouse from falling. The test started shortly after the mouse held onto the metal net, and the time when the mouse fell off was recorded four times with at least 5 min intervals between each test.

#### Beam-walk test

The beam-walk test was carried out using horizontal wooden beams that were 12 and 22 mm in diameter to assess coordination capabilities. The mice received 3 training sessions, and the time the mice passed the 1 m long wooden beam was recorded. The test was repeated four times with at least 5 min between each test.

### The enzymatic activities of aconitase and complexes I and II

The aconitase activity was measured according to a previous method [33]. The activities of complexes I and II were measured following the manufacturer’s protocols. Purchase information is as follows: Complex I from Abcam and Complex II from Comin Biotechnology Co. (Suzhou, Jiangsu, China).

### Detection of SOD and catalase enzyme activities

The activities of SOD and catalase were measured following the manufacturer’s protocols. Purchase information was as follows: total SOD assay kit from Beyotime Biotechnology (Shanghai, China) and catalase assay kit from Nanjing Jiancheng Bioengineering Institute (Nanjing, China).

### Detection of malondialdehyde (MDA) and ATP content

The MDA content was detected according to the manufacturer’s protocol (Beyotime Biotechnology). The ATP level in tissues or cells was measured using the ATP Assay Kit (Beyotime Biotechnology).

### Histological assays

The tissue was embedded in paraffin and cut into 2 to 7 μm thick sections by a rotation microtome.

#### H&E staining

Hematoxylin–eosin staining (H&E staining) was performed as previously reported [33].

#### Immunofluorescence

The obtained tissue Section (4 μm thickness) were incubated with primary antibodies against DCFH-DA (Beyotime Biotechnology, catalog number S0033S) or NeuN (Proteintech, catalog number 26975-1-AP) overnight at 4 °C. The sections were further coincubated with rabbit fluorescence secondary antibody for 120 min at room temperature. Finally, the stained sections were observed and photographed by confocal microscopy.

#### Prussian blue staining

Raw 264.7 cells were cultured with 10 μM IONPs for 24 h and then treated with 2 μM curcumin for 24 h. Freshly prepared 5% potassium ferrocyanide and 5% hydrochloric acid were used to detect the level of iron by Prussian blue staining. Photograph analysis was performed after dyeing the nucleus.

### Western blot analysis and antibody information

Mouse tissues were lysed by RIPA lysis buffer (Beyotime, Shanghai, China), and cells were lysed by lysis buffer (1% NP-40, 40 mM Tris, 4 M NaCl, with protease inhibitor). 10–50 µg proteins per lane were tested by SDS–PAGE gel. Antibody information is as follows: anti-Ndufs1 (Proteintech, cat# 12444-1-AP), anti-SDHB (Abcam; catalog number 178423), anti-UQCRFS1 (Proteintech; catalog number 18443–1-AP), anti-β-Actin (Bioworld; catalog number AP0060), anti-Tubulin (Invitrogen; catalog number A11126), anti-GAPDH (Proteintech; catalog number 60004–1-Ig), anti-Rabbit Aco2 (Proteintech, catalog number 11134–1-AP), and anti-Rabbit NFS1 (Proteintech, catalog number 15370-1-AP). Antibodies against FXN and ISCU were validated in previous studies [32].

### Statistical analysis

All experiments were performed using at least three independent biological replicates. Values were presented as the mean ± SEM. For two-group comparisons, an unpaired two-tailed Student’s t test was used. Differences with p < 0.05 were considered statistically significant. Statistical tests were performed with GraphPad Prism 5.01 and 8.4.2.

## Results

### Preparation and characterization of Cur@SF NPs

**Fig. 1:**
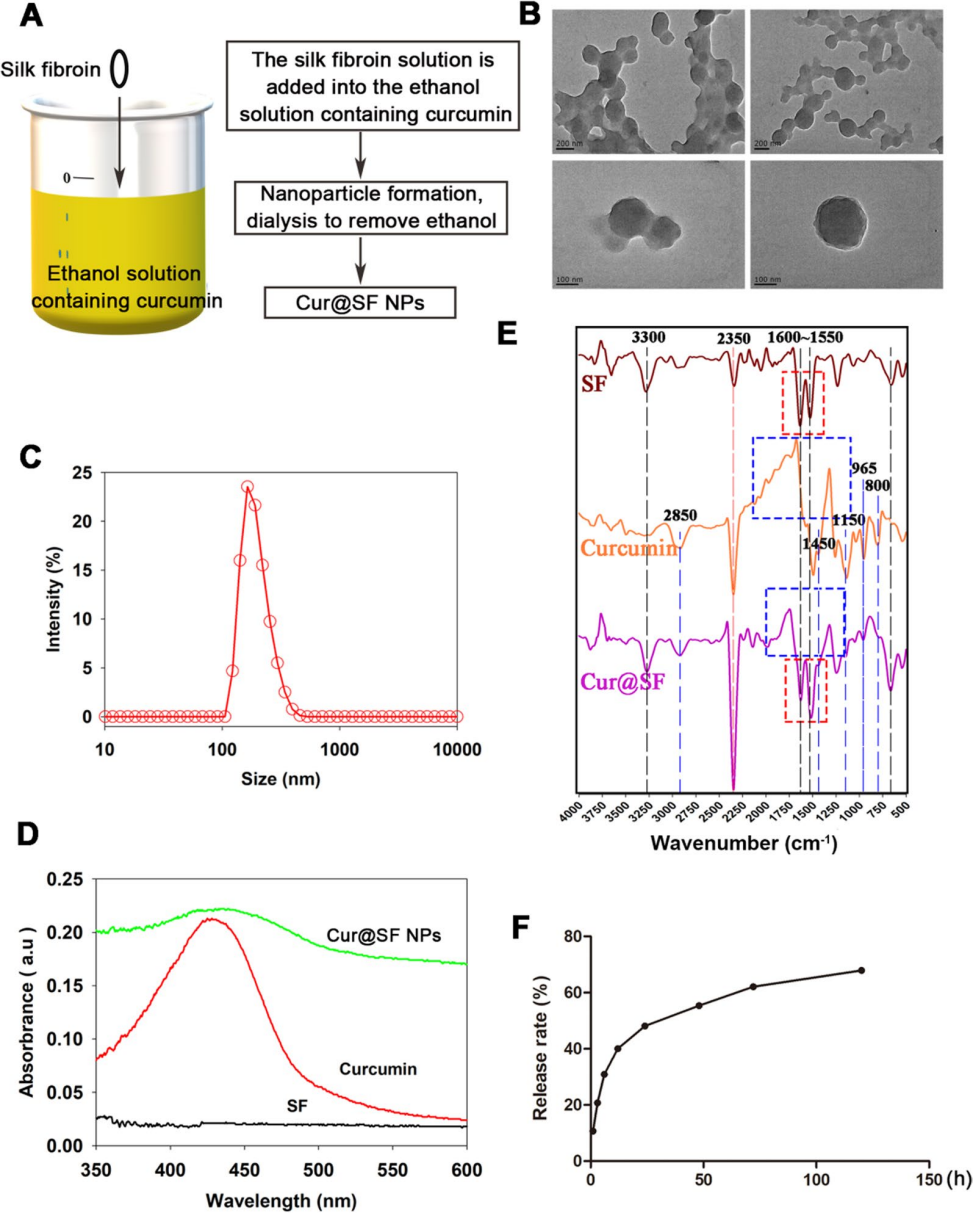
Preparation and characterization of Cur@SF NPs. **A** Preparation process of silk fibroin-loaded curcumin (Cur@SF NPs). **B** Scanning electron microscopy (SEM) image of Cur@SF NPs at two magnifications (scale bars: 200 nm and 100 nm). **C** Hydrodynamic size distribution of Cur@SF NPs. **D** Ultraviolet and visible spectrophotometry (UV–Vis) spectra showing the characteristic absorption of curcumin in Cur@SF NPs. **E** Fourier transform infrared spectroscopy (FTIR) of SF, curcumin and Cur@SF NPs. The spectral peaks at 1010 cm^−1^ and 1046 cm^−1^ were assigned to the -C–O–C- vibration of curcumin. The anti-symmetric stretching of the C–O–C from curcumin is at approximately 1150 cm^−1^. The characteristic absorption peak of SF was observed at 3300 cm^−1^, which represented the stretching vibration of the peptide bond (-CONH-) of SF. Specific peaks were also observed at 1600 cm^−1^ (amide I, C-O) and 1550 cm^−1^ (amide II, N–H), which represented β-sheets of SF. The carbonyl peak of curcumin and the amide peak of SF may partially overlap, which are all attributed to the strong absorption at approximately 1500 cm^−1^. **F** Determination of the release of curcumin from Cur@SF NPs in type XIV collagenase solution by detecting curcumin content within 120 h

As displayed in Fig. 1A, curcumin was dissolved in anhydrous ethanol, and the conformation of silk fibroin changed in alcohol solution, forming cohesive nanospheres and encapsulating dissolved curcumin. SEM showed the spherical shape of Cur@SF NPs with an average particle size of approximately 150 nm (Fig. 1B). The nanoparticles exhibited good dispersibility in aqueous solvent, and their hydrodynamic size distribution was between 100 and 360 nm according to dynamic light scattering (DLS) (Fig. 1C). In addition, curcumin alone in alcohol solution and assembled as Cur@SF NPs both presented visible light absorption at 435 nm (Fig. 1D). FTIR spectra showed the characteristic absorption of the components of Cur@SF NPs (Fig. 1E). The slow-release characteristics of Cur@SF NPs verified that curcumin released from nanoparticles was time-dependent following protease XIV treatment (Fig. 1F).

**Fig. 2:**
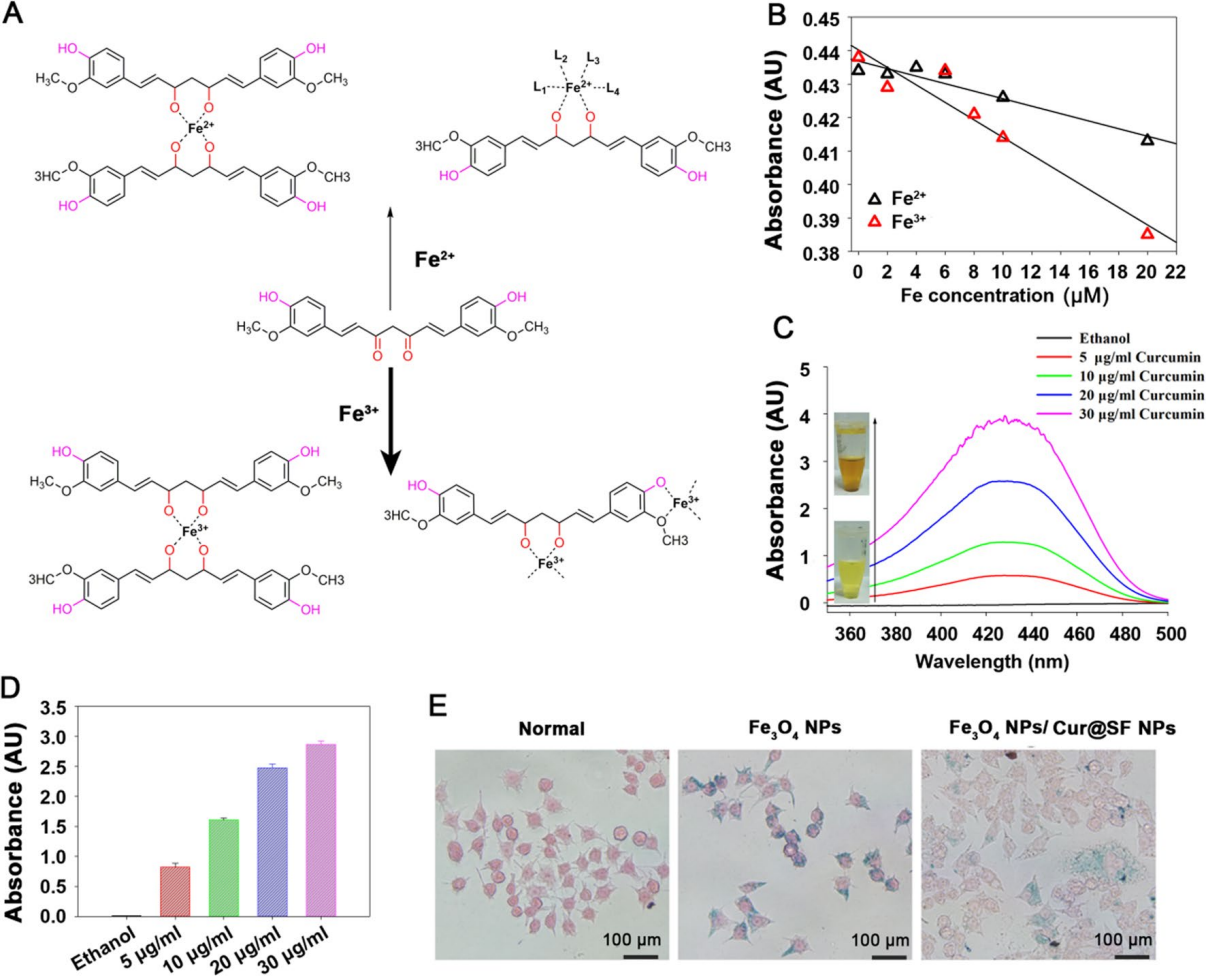
Iron chelating ability of curcumin in vitro. **A**, **B** The chemical structure of curcumin and the binding site where curcumin binds to iron ions (Fe^2+^ and Fe^3+^). **C**, **D** UV–Vis spectrum, peaked at 435 nm, to demonstrate iron chelation by curcumin in a concentration-dependent manner. Ultrasmall iron oxide nanoparticles (IONPs, 10 μM iron) were used as an iron source, and the absorption at 435 nm was quantified in D. **E** Prussian blue staining was used to reveal Fe accumulation after IONP or IONP + Cur@SF NPs treatment in RAW 264.7 cells

Due to the dicarbonyl structure, curcumin can be complexed by Fe^2+^ and Fe^3+^, while the methoxy and phenolic hydroxyl structures on the benzene ring exhibited the ability to preferentially chelate Fe^3+^ (Fig. 2A) [34–36]. We evaluated the complexation ability of curcumin with iron by measuring the decrease in absorbance at 435 nm. The data confirmed the preference of curcumin for Fe^3+^, other than Fe^2+^ (Fig. 2B), in a concentration-dependent manner (Fig. 2C, D). We then tested the capacity of iron chelation by curcumin in cells. Ultrasmall iron oxide nanoparticles (IONPs, Fe_3_O_4_ NPs) were used to mimic aggregated cellular ferric acid, which is present in cardiomyocytes of FRDA patients. Fe_3_O_4_ NPs were prepared according to the previous method [37, 38]. The particle sizes were determined in the range of 10–20 nm in diameter by transmission electronical microscopy (TEM) (Additional file 1: Fig. S2A), and the dynamic light scattering (DLS) confirmed that the hydrodynamic size of Fe_3_O_4_ NPs was 10–80 nm (Additional file 1: Fig. S2B), demonstrating the successful preparation of Fe_3_O_4_ NPs. As shown in Fig. 2E and Additional file 1: Fig. S3, Cur@SF NPs were able to efficiently chelate iron from IONPs to prevent their accumulation in RAW26.4 cells. The quantification result was shown in Fig. S3. We also plotted the iron chelation curves of curcumin, SF and Cur@SF NPs, and the result showed that SF was not able to chelate iron, but curcumin and Cur@SF NPs directly chelated iron (Additional file 1: Fig. S4).

### Cur@SF NPs reduce the levels of oxidative stress in fibroblasts derived from FRDA patients

**Fig. 3:**
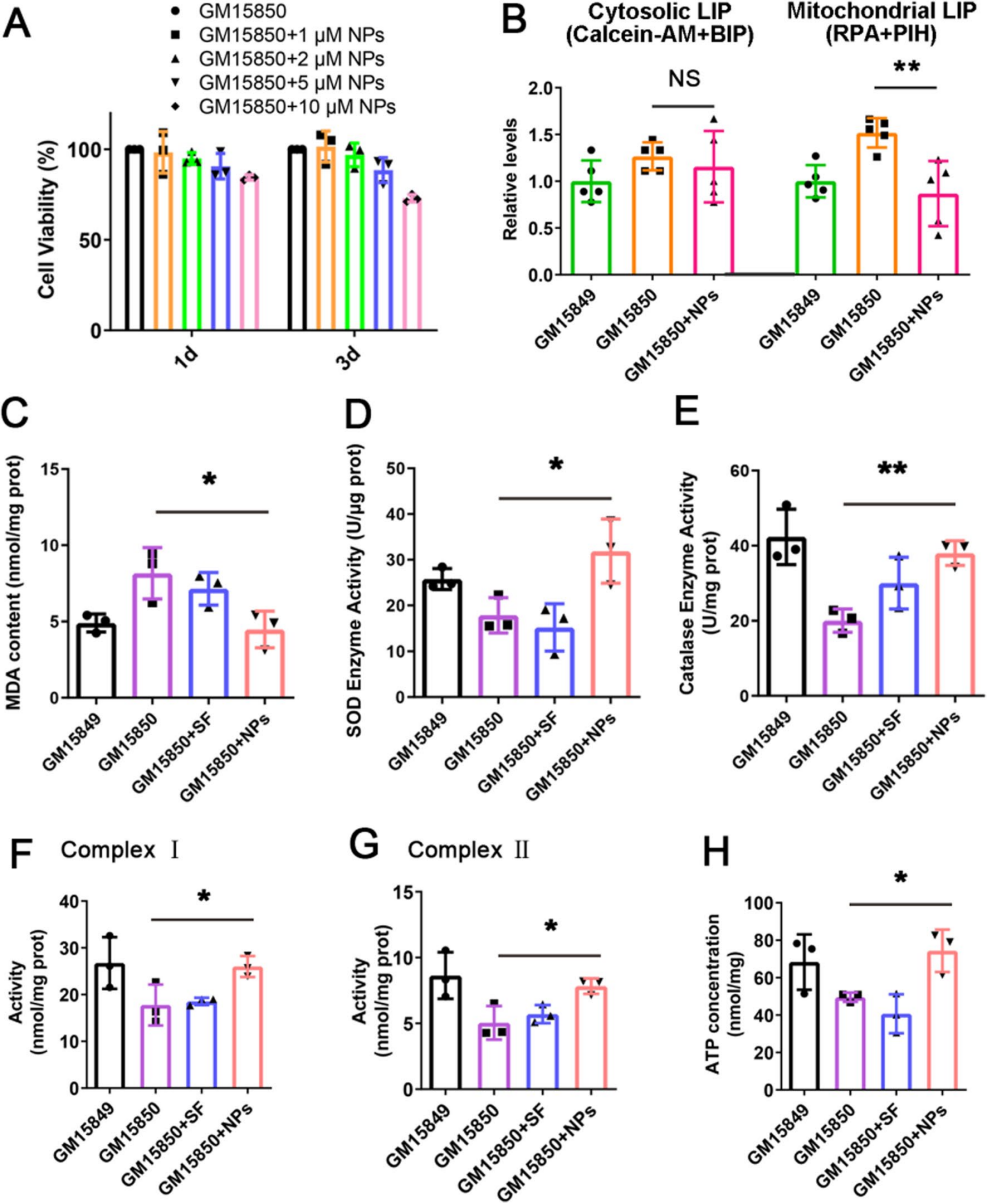
Iron chelation and antioxidant effect of Cur@SF NPs in vitro. **A** The relative value of CCK-8 in lymphocytes (GM15850) derived from FRDA patients treated with Cur@SF NPs (NPs) at different concentrations for 1 or 3 days. **B** The relative levels of mitochondrial LIP (labile iron pool) measured with RPA along with pyridoxal isonicotinoyl hydrazine (PIH) following drug treatment in lymphocytes GM15849 and GM15850. The relative levels of cytosolic LIP measured by calcein-AM along with 2,2′-bipyridyl (BIP) (for details, see “Materials and methods” Section). **C** The content of malondialdehyde (MDA). **D**, **E** The enzymatic activities of SOD and catalase. **F, G** The activities of mitochondrial complexes I and II. **H** Cellular ATP content. (*, p < 0.05; **, p < 0.01, GM15850 + NPs vs. GM15850)

The antioxidative effect of curcumin is widely implicated in the improvement of neurodegenerative diseases. To see the effects in FRDA models, we first evaluated the safety of Cur@SF NPs in lymphocytes (GM15850, Coriell) derived from a FRDA patient. The concentration (of curcumin) was optimized to be 1–2 μM without any harm to viability after treatment for 1 to 3 days (Fig. 3A). Interestingly, the iron content of the labile iron pool was decreased in mitochondria but not in the cytosol (Fig. 3B), indicating that the curcumin released from Cur@SF NPs modulated iron homeostasis, at least partially, by chelating mitochondrial iron.

Moreover, we found here that Cur@SF NPs (1 μM curcumin) significantly reduced the levels of oxidative stress in GM15850 cells, as indicated by the diminished content of malondialdehyde (MDA, the lipid peroxidation index) and the increased activities of antioxidant enzymes (SOD and catalase) (Fig. 3C–E). To test whether dysfunctional mitochondria were rescued in GM15850 cells, we measured mitochondrial complex activities and ATP supply. As shown in Fig. 3F–H, the activities of mitochondrial complexes I and II and ATP production were both significantly increased. These data proved the effectiveness of Cur@SF NPs in improving mitochondrial function, very likely through antioxidation and iron chelation by released curcumin.

### Cur@SF NPs restored the behavioral scores of mice

**Fig. 4:**
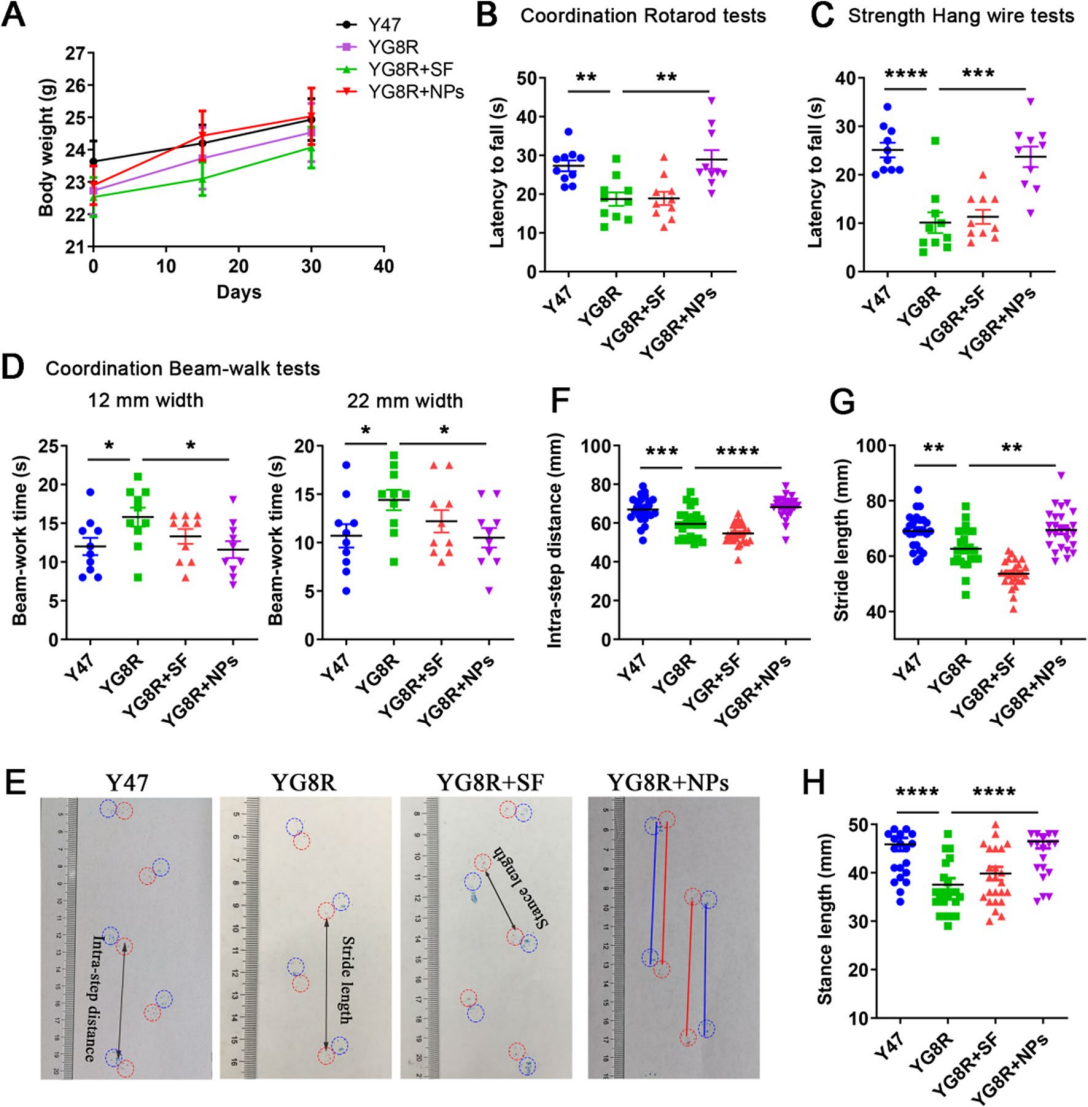
Cur@SF NPs increase the behavioral scores of FRDA mice. YG8R mice were intraperitoneally injected with Cur@SF NPs every five days for one month when the mice were 4 months old, and then the behavioral changes were assessed. Y47: KOKI mice containing human *FXN* as wild type; YG8R: KOKI mice containing human *FXN* with (*GAA*)n insert as FRDA model; YG8R + SF: YG8R with silk fibroin administration as a drug delivery vehicle; YG8R + NPs: YG8R mice treated with Cur@SF NPs. **A** The body weight of mice. The mice were weighed every 15 days during drug administration (n = 10/group). The initial age was 4 months old. **B** Coordination beam-walk tests. **C** Coordination rotarod tests. **D** Strength hang wire tests. **E**–**H** Gait tests. The front foot was dyed red, and the rear foot was dyed blue. (*p < 0.05; **p < 0.01; ***p < 0.001; ****,p < 0.0001, YG8R + NPs vs. YG8R)

Curcumin has been widely studied in other neurodegenerative diseases [39–41]. Given that curcumin exhibits satisfying iron chelating capacity and antioxidative capacity, we tried to apply Cur@SF NPs in the treatment of FRDA mice with *FXN* deficiency, in which mice were developed by Dr. Mark Pook and his colleagues, and Y47 was used as a control [42]. Cur@SF NPs were injected intraperitoneally into 4-month-old YG8R mice once every 5 days for one month. Then, the weight was recorded, and no significant difference was observed between the treated and untreated groups (Fig. 4A). However, the behavior assays showed that the coordination and strength were both improved, revealed by longer latency to fall in the rotarod tests (Fig. 4B), by shorter time to cross in the beam-walk tests (Fig. 4C) and longer time to hold in hang wire tests (Fig. 4D) in treated YG8R mice than in untreated YG8R mice. Simultaneously, gait tests were performed, and the results showed a similar outcome: the intrastep distance, stride length, and stance length were all significantly increased in Cur@SF NP treated mice compared with untreated mice to levels very close to those in Y47 control mice (Fig. 4E–H). These results demonstrated that Cur@SF NPs were effective in restoring the behavioral dysfunction of FRDA mice.

### Cur@SF NPs restore mitochondrial morphology and function in FRDA mice

**Fig. 5:**
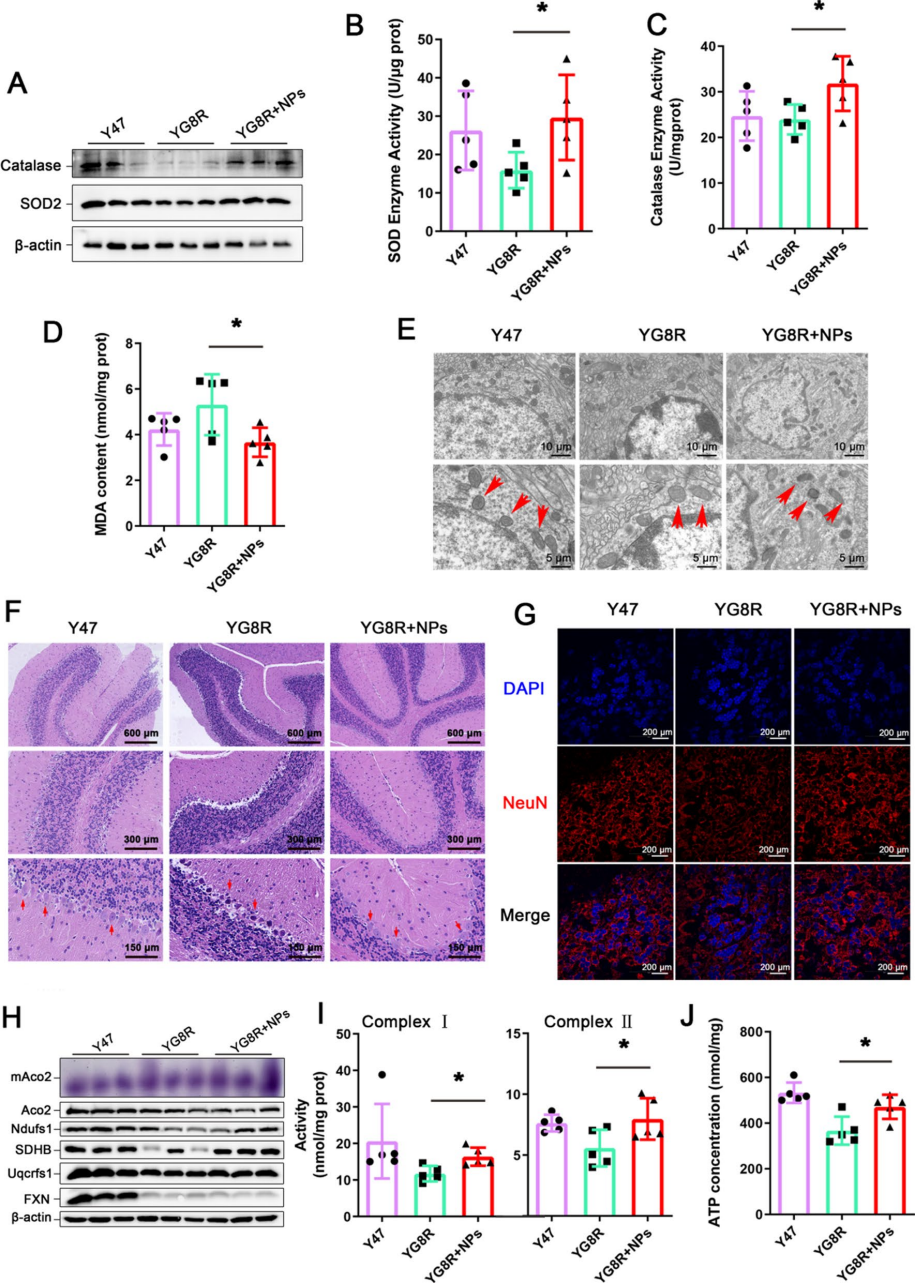
Cur@SF NPs reduce oxidative stress and restore the mitochondrial function of the cerebellum in YG8R mice. All samples were from tissue cerebellum. **A** Detection of the catalase and SOD2 protein levels. **B**–**D** The enzymatic activities of SOD, catalase and MDA content in the cerebellum after Cur@SF NP treatment (n = 5). **E** TEM results of cerebellar tissue to observe mitochondrial morphology. **F** H&E staining to evaluate the recovery of morphology and array of Purkinje neurons as indicated by the red arrows. **G** Immunofluorescent staining of NeuN to determine the number of neurons. **H** Detection of mitochondrial aconitase activity (purple) and mitochondrial protein levels. mAco2: mitochondrial aconitase (Aco2) activity (purple); Ndufs1, SDHB, and Uqcrfs1: mitochondrial complex I/II/III subunits. **I** The activities of mitochondrial complexes I and II. **J** ATP content. (*p < 0.05; *NS* no significance; YG8R + NPs vs. YG8R, n = 5/group)

Nano-assembly has significant advantages in scavenging free radicals in living organisms [43, 44]. Curcumin has been shown to activate the nuclear factor E2-related factor 2 (Nrf2) pathway, triggering cellular protection against oxidative injury [45]. As two members of the Nrf2 regulon, the protein levels of catalase and SOD2 were upregulated by curcumin [46, 47], which was exactly observed in our study (Fig. 5A), while the activities of antioxidative enzymes significantly increased (Fig. 5B, C). As a result, the MDA content decreased (Fig. 5D).

To further evaluate whether the excellent property of Cur@SF NPs against oxidative stress would benefit mitochondria, we analyzed the morphology and function of mitochondria [13]. The mitochondria in the cerebellum were imaged by TEM. Compared to the control mice Y47, mitochondria from FRDA mice YG8R exhibited a typical low density of cristae, but presented an improved density of cristae post treatment with Cur@SF NPs (Fig. 5E). Histological assays through H&E staining further demonstrated that Purkinje neurons in the edge around the dentate gyrus of the cerebellum shrank and were dark-stained but were full and round and light-stained after Cur@SF NP treatment (Fig. 5F). Meanwhile, the restorative neuron number in the cerebellum could be observed by NeuN immunolabeling in the treated YG8R mice (Fig. 5G).

To track the relevant changes at the molecular level, we extracted proteins from the cerebellum and detected the expression levels of mitochondrial complex-related proteins (Ndufs1, SDHB, Uqcrfs1) and the activity of mitochondrial aconitase (mAco2). The upregulation of the proteins and increased mAco2 activity further confirmed the recovery of mitochondrial function in cerebellar tissue of the treated YG8R mice (Fig. 5H). Consequently, mitochondrial function was improved, as revealed by the elevated activities of mitochondrial complexes (I and II) and ATP content (Fig. 5I, J).

### Cur@SF NPs eliminate cardiac iron accumulation and improve cardiomyocyte hypertrophy

**Fig. 6:**
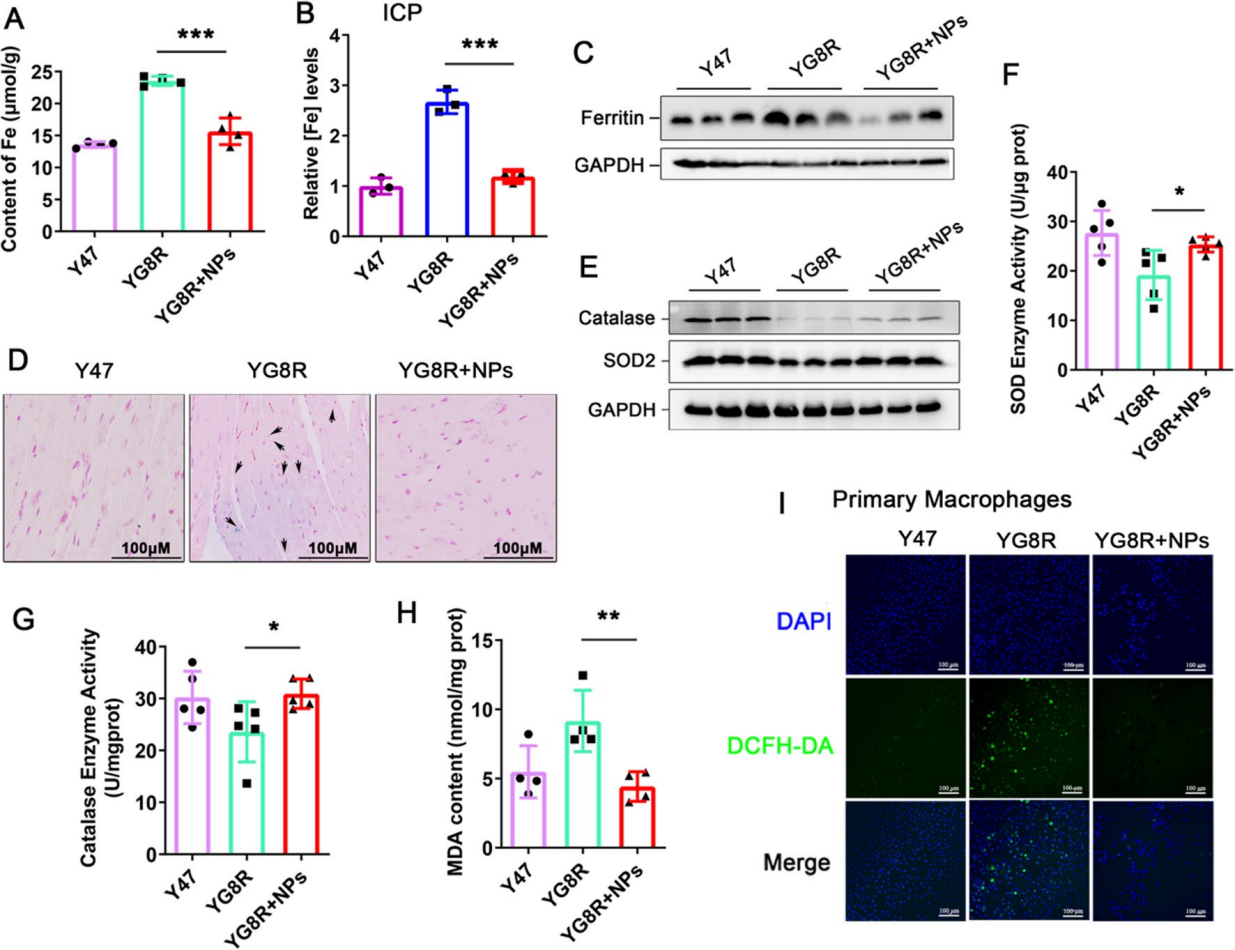
Cur@SF NPs eliminate cardiac iron accumulation and reduce oxidative stress levels. All samples were from tissue heart. **A** Ferrozine assays for iron content. **B** The relative levels of iron detected with ICP. **C** Detection of the ferritin protein level. **D** Prussian staining to detect iron in cardiomyocytes. **E** Detection of the catalase and SOD2 protein levels. **F**–**H** The enzymatic activities of SOD, catalase and MDA content in tissue hearts after Cur@SF NP treatment (n = 5). **I** Primary macrophages were stained with the green fluorescent dye DCFH-DA after 2 μM Cur@SF NPs treatment for 24 h. (*p < 0.05; **p < 0.01; ***p < 0.001, YG8R + NPs vs. YG8R)

The heart is another seriously affected tissue for FRDA patients, and approximately 60% of patients die from lethal congestive heart failure and supraventricular arrhythmias [9]. Given that iron retention is the main cause of myocardial dysfunction in FRDA patients and curcumin can chelate iron, we further examined the iron levels in myocardium after Cur@SF NP administration. The iron content was significantly lower in the hearts of treated YG8R mice than in the hearts of untreated YG8R mice, as revealed by ferrozine assays and ICP (Fig. 6A, B). In consistence with this, ferritin expression decreased following treatment with Cur@SF NPs (Fig. 6C). Prussian blue staining of the sliced tissue further confirmed iron deposition in the hearts of YG8R mice and was hardly detectable after treatment (Fig. 6D). Again, curcumin increased the protein levels and enzymatic activities of catalase and SOD2 (Fig. 6E–G). The results were consistent with those in the cerebellum accompanied by decreased MDA content after treatment (Fig. 6H). To directly examine the antioxidative capacity of Cur@SF NPs, we isolated primary macrophages from YG8R mice. DCFH-DA fluorescence showed the reduced oxidative stress after Cur@SF NPs treatment (Fig. 6I), confirming the antioxidative efficacy of Cur@SF NPs.

**Fig. 7:**
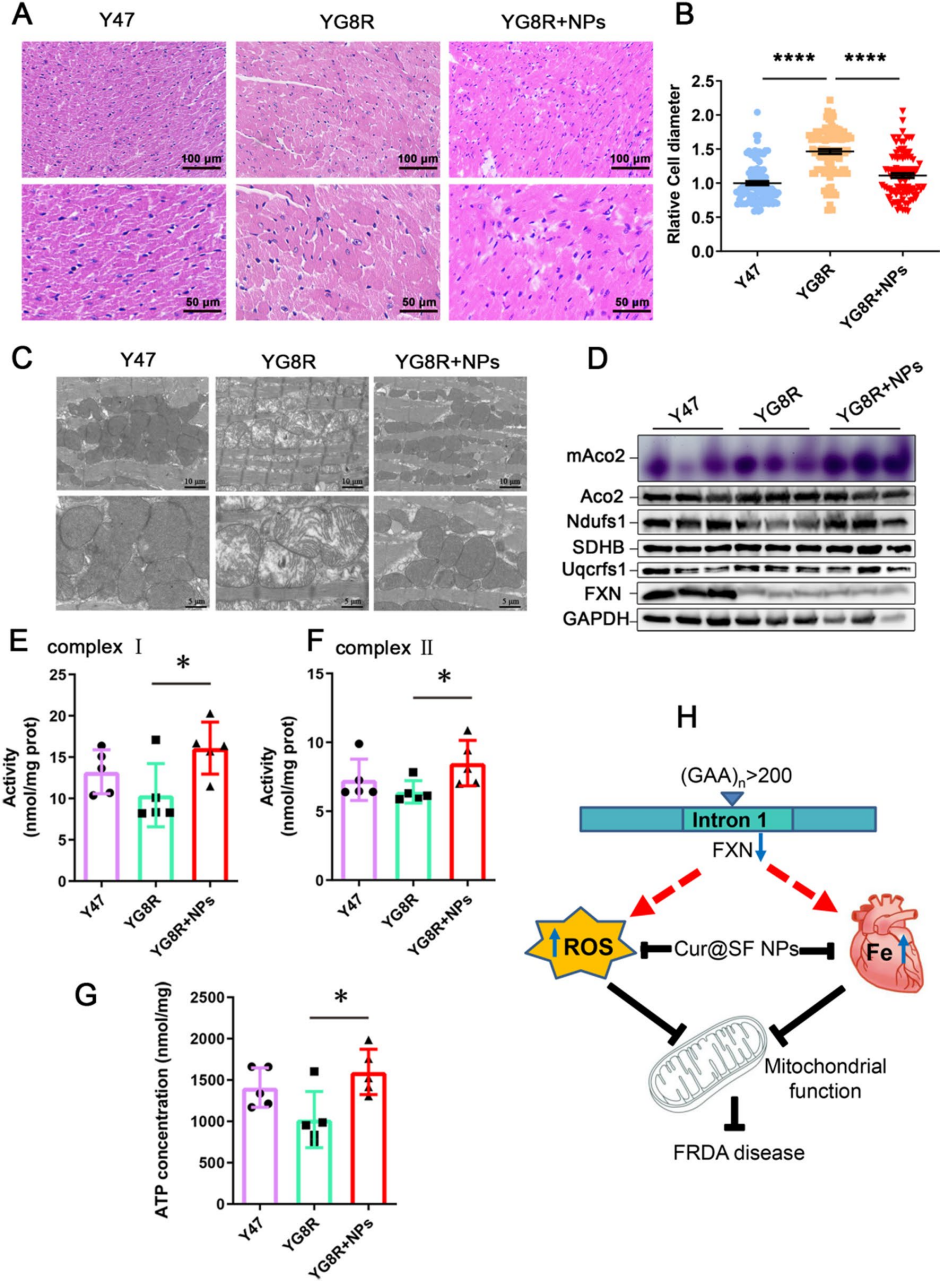
Cur@SF NPs significantly improved myocardial function.** A**, **B** H&E staining to evaluate the improvement of cardiomyocyte hypertrophy by counting the diameter of cardiomyocytes. **C** TEM results to observe the mitochondrial morphology and internal ultrastructure. **D** Detection of aconitase activity (purple) and protein levels of mitochondrial proteins. mAco2: mitochondrial aconitase (Aco2) activity. **E**, **F** The activities of mitochondrial complexes I and II. **G** ATP content. **H** A schematic diagram depicting Cur@SF NPs alleviating FRDA through synergistic iron chelation and antioxidation. (*p < 0.05; ****p < 0.0001, YG8R + NPs vs. YG8R, n = 5/group)

FXN deficiency-induced iron overload is strongly associated with myocardial hypertrophy. After H&E staining, we counted the cell diameter to evaluate cardiomyocyte hypertrophy. The average diameter of cardiomyocytes was larger in YG8R mice than in Y47 mice but significantly reduced compared with untreated mice (P < 0.001) to be close to that in Y47 mice (Fig. 7A, B). The low density of mitochondrial cristae remarkably became dense in YG8R heart following Cur@SF NP treatment, as revealed by TEM (Fig. 7C). Interestingly, we found that the sizes of the mitochondria, in general, were larger in the cardiomyocytes of YG8R than in those of Y47, which was diminished in the treated group (Fig. 7C). Total proteins were extracted from the heart and used to detect the expression levels of Ndufs1, SDHB, and Uqcrfs1 and the activity of mAco2. Only Ndufs1 expression was significantly increased, although not SDHB and Uqcrfs1, which remained constant under the three conditions. However, the enzymatic activity of mAco2 was greatly higher in YG8R mice after treatment, even better than that in Y47 mice (Fig. 7D). Accordingly, ATP content and the activities of mitochondrial complexes (I and II) were restored in the hearts of the treated YG8R mice (Fig. 7E–G). Taken together, Cur@SF NP treatment is greatly beneficial to the recovery of mitochondrial morphology and function in FRDA mice.

## Discussion

Mitochondrial dysfunction is the main cause of nerve and cardiomyocyte degeneration in FRDA patients. The deficit of frataxin protein induces an insufficiency of iron-sulfur cluster synthesis and supply, from which iron overload and oxidative stress directly result [48]. To date, there is no effective therapy to slow down or/and reverse FRDA neuropathy [49, 50], but with promise reported very recently [51].

Increasing FXN protein levels through upregulation by drugs or gene therapies would bring great hope if there are no severe side effects [52]. Before that, removal of symptom-causing factors, such as iron retention and oxidative stress, would be an alternative for FRDA treatment. Iron chelators (deferoxamine or deferiprone) and/or antioxidants (vitamin E, Vc, coenzyme Q10, Nrf2 activator, and so on) are in trials, although some of them are still ongoing and some of them fail (see pipeline at www.curefa.org and refs [14, 15]). Currently, the Nrf2 activator RTA-408 is the most promising activator in phase III clinical trials and showed positive results [51]. Before a final satisfying therapy was available, a compound, curcumin, might be a worthy attempt. Curcumin has been demonstrated to induce stabilization of the Nrf2 protein through Keap1 cysteine modification [53]. Nrf2 is a master transcription factor that responds against oxidative stress. Here, we assembled curcumin-loaded slowly released nanoparticles, which provided benefits, very likely through a similar mechanism by activating Nrf2 to defense against oxidative stress overall and by the capacity of iron chelation, particularly in the heart to protect mitochondria, therefore relieving FRDA (Fig. 7H).

Although we did not detect how much Cur@SF NPs crossed the blood brain barrier (BBB), the concentration of curcumin in this study was chosen to be 1 μM in vitro and 150 mg/kg/5 days in vivo, and curcumin administration provided significant protection against mitochondrial dysfunction, indicating increased bioavailability and capability to cross the BBB. Another attempt was to use low-intensity focused ultrasound to open the BBB to facilitate curcumin delivery into the deep brain of PD mice and improve behavior [54, 55]. In addition, to increase the drug delivery efficiency, our team tried to use near-infrared light-induced drug release technology [56] and radiofrequency ablation drug release technology [57] to maximize the bioavailability of curcumin.

Interestingly, curcumin has been demonstrated to be able to inhibit protein aggregation by directly binding to unfolded proteins [58], which is considered to be the key pathogenesis of AD and PD. Notably, early intervention by curcumin could reduce the progression of AD-like pathological outcomes more significantly [59]. We only tested Cur@SF NPs at 4 months age of FRDA mice and do not know if the satisfying efficacy of Cur@SF NPs would be reached at late stage of FRDA mice.

In summary, we have verified in vitro and in vivo that Cur@SF NPs are able to significantly improve mitochondrial morphology and function in FRDA models. The improvement of the preclinical manifestation is astonishing that Cur@SF NPs took action in reconstructing cerebellar neurons and improving myocardial hypertrophy, suggesting a promising treatment for FRDA.

## Supplementary Information

**Additional file 1. Fig. S1.** A calibration curve of curcumin with known concentrations. The absorption value was recorded at 435 nm. **Fig. S2.** Characterization of Fe3O4 nanoparticles (Fe3O4 NPs). **Fig. S3.** The quantified result of Fig. 2E. **Fig. S4.** The curves of iron chelating capacity of curcumin and Cur@SF NPs.

## Data Availability

All data generated or analyzed during this study are included in this article.
