# Supplementary material for: Cur@SF NPs alleviate Friedreich’s ataxia in a mouse model through synergistic iron chelation and antioxidation
Source: J Nanobiotechnology. 2022 Mar 9;20:118. doi: 10.1186/s12951-022-01333-9 (PMC8905737; doi:10.1186/s12951-022-01333-9)
Supplement: Supplementary file 1 — Additional file 1. Fig. S1. A calibration curve of curcumin with known concentrations. The absorption value was recorded at 435 nm. Fig. S2. Characterization of Fe3O4 nanoparticles (Fe3O4 NPs). Fig. S3. The quantified result of Fig. 2E. Fig. S4. The curves of iron chelating capacity of curcumin and Cur@SF NPs. [file 12951_2022_1333_MOESM1_ESM.docx]

**Cur@SF NPs Alleviate Friedreich’s Ataxia in a Mouse Model** **through Synergistic Iron Chelation and Antioxidation**

**Li Xu^1,2^, Zichen Sun^1,2^, Zhiyao Xing^1,2^, Yutong Liu^1,2^, Hongting Zhao^1,2^, Zhongmin Tang^3^, Yu Luo^4^, Shuangying Hao^5*^, Kuanyu Li^1,2*^**

^1^State Key Laboratory of Pharmaceutical Biotechnology, Division of Iron Metabolism and Mitochondrial Function, Medical School of Nanjing University, Nanjing 210093, China.

^2^Jiangsu Key Laboratory of Molecular Medicine, Medical School of Nanjing University, Nanjing 210093, China

^3^Shanghai Institute of Ceramics, Chinese Academy of Sciences, Shanghai 200050, China

^4^Shanghai Engineering Technology Research Center for Pharmaceutical Intelligent Equipment, Shanghai Frontiers Science Research Center for Druggability of Cardiovascular noncoding RNA, Institute for Frontier Medical Technology, College of Chemistry and Chemical Engineering, Shanghai University of Engineering Science, Shanghai, 201620, China

^5^School of Medicine, Henan Polytechnic University, Jiaozuo, 454003, Henan Province, China

**^*^ Correspondence:**

**Kuanyu Li**

[likuanyu@nju.edu.cn](mailto:likuanyu@nju.edu.cn)

**Shuangying Hao**

[shuangying9088@hpu.edu.cn](mailto:shuangying9088@hpu.edu.cn)


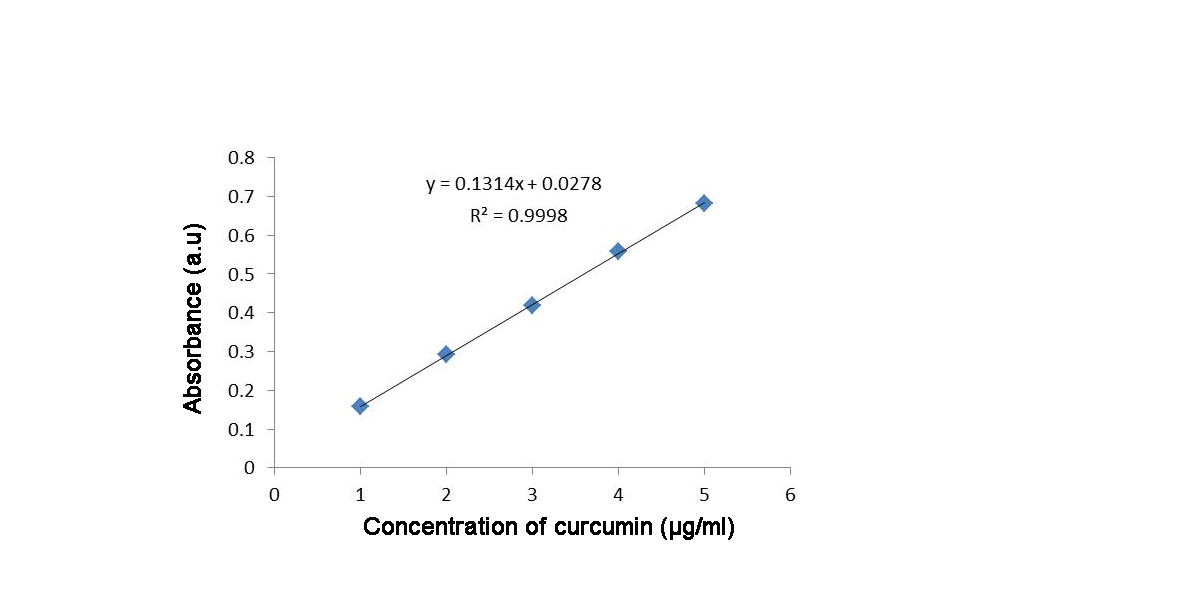


Fig. S1. **A calibration curve of curcumin with known concentrations.** The absorption value was recorded at 435 nm.


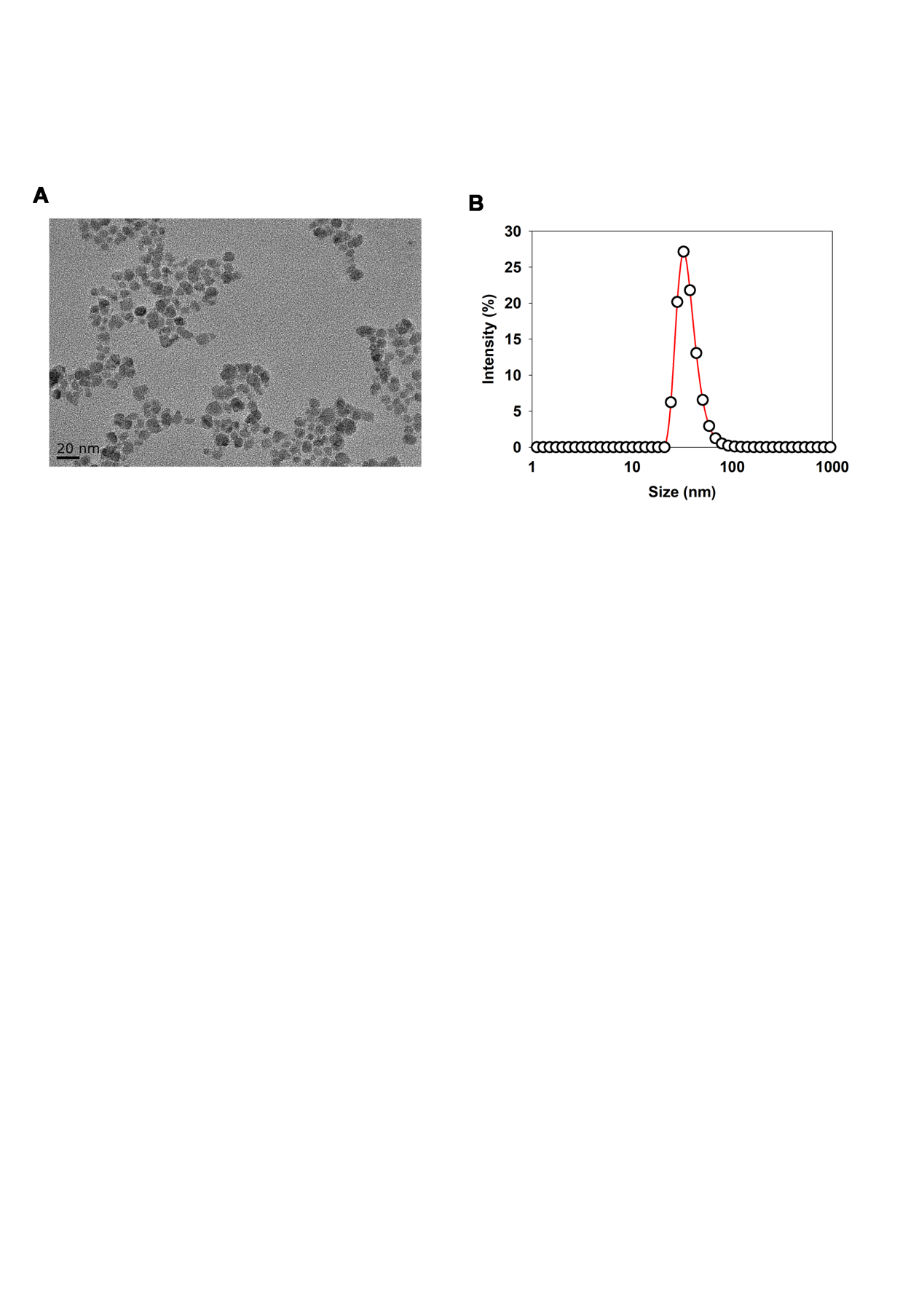


Fig. S2. **Characterization of Fe_3_O_4_ nanoparticles (****Fe_3_O_4_ NPs). A** Transmission electron microscopy (TEM) image of Fe_3_O_4_ NPs (scale bars: 20 nm). **B** Hydrodynamic size distribution of Fe_3_O_4_ NPs, revealed by dynamic light scattering (DLS).


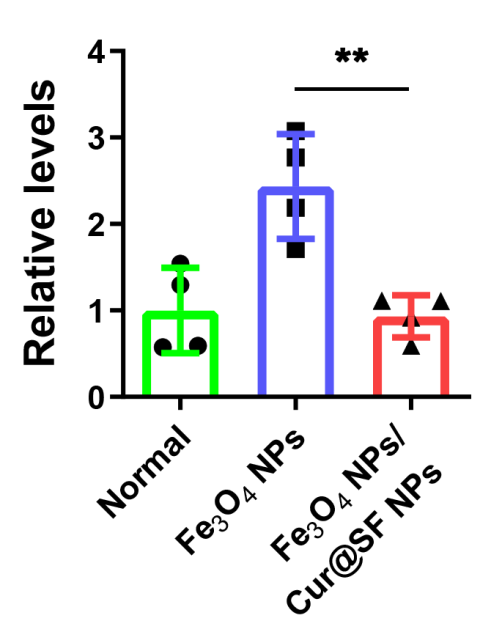


Fig. S3. **The quantified result of Fig. 2E.** Prussian Blue staining was used to reveal iron accumulation after IONP or IONP+Cur@SF NPs treatment in RAW 264.7 cells.


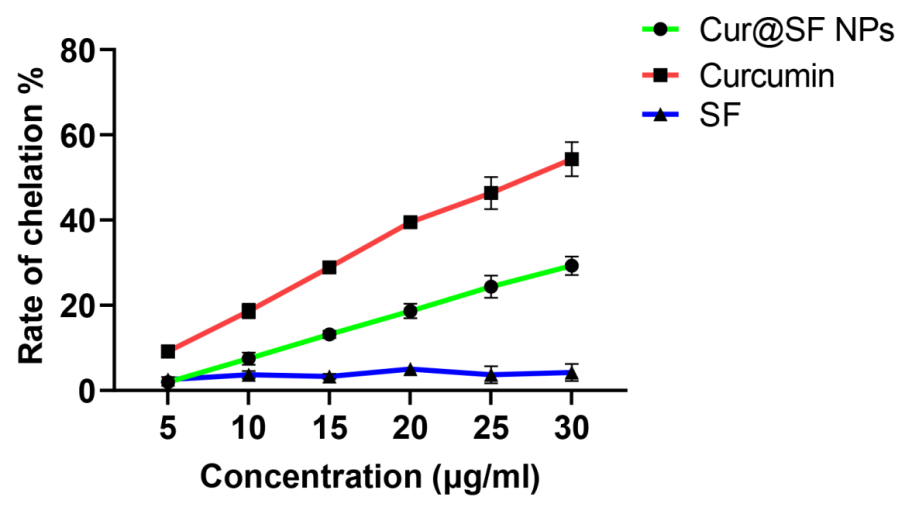


Fig. S4. **The curves of iron chelating capacity of curcumin and Cur@SF NPs.** SF nanoparticles were made (see **Materials and Methods**). Curcumin, SF nanoparticles and Cur@SF NPs were all frozen-dried into powders and then formulated into solutions at concentrations of 5, 10, 15, 20, 25 and 30 μg/ml. FeCl_3_ (200 μL of 50 μM) was added to 1 ml of the above three different solutions at 4℃ for 24 h with rotation. The supernatant was collected by centrifugation. The iron content in the supernatant was determined by Ferrozine iron assay (in **Materials and Methods**). The decreased level of iron is calculated as the chelated iron.
